# Flying Drosophila show sex-specific attraction to fly-labelled food

**DOI:** 10.1038/s41598-019-51351-1

**Published:** 2019-10-18

**Authors:** Laurie Cazalé-Debat, Benjamin Houot, Jean-Pierre Farine, Claude Everaerts, Jean-François Ferveur

**Affiliations:** 1 Centre des Sciences du Go û t et de l’Alimentation, AgroSup, UMR6265 CNRS, UMR1324 INRA, Université de Bourgogne Franche-Comté, 6, Bd Gabriel, 21000 Dijon, France; 2 Present Address: Laboratoire de Biologie Fonctionnelle et Adaptative, UMR8251, Université Paris Diderot - Paris 7/CNRS Equipe Processus Dégénératifs, Stress et Vieillissement Bâtiment Buffon, 4, rue Marie Andrée Lagroua Weill Halle, 75013 Paris, France

**Keywords:** Chemical ecology, Behavioural ecology

## Abstract

Animals searching for food and sexual partners often use odourant mixtures combining food-derived molecules and pheromones. For orientation, the vinegar fly *Drosophila melanogaster* uses three types of chemical cues: (*i*) the male volatile pheromone 11-*cis*-vaccenyl acetate (*c*VA), (*ii*) sex-specific cuticular hydrocarbons (CHs; and CH-derived compounds), and (*iii*) food-derived molecules resulting from microbiota activity. To evaluate the effects of these chemicals on odour-tracking behaviour, we tested Drosophila individuals in a wind tunnel. Upwind flight and food preference were measured in individual control males and females presented with a choice of two food sources labelled by fly lines producing varying amounts of CHs and/or *c*VA. The flies originated from different species or strains, or their microbiota was manipulated. We found that (*i*) fly-labelled food could attract—but never repel—flies; (*ii*) the landing frequency on fly-labelled food was positively correlated with an increased flight duration; (*iii*) male—but not female or non-sex-specific—CHs tended to increase the landing frequency on fly-labelled food; (*iv*) *c*VA increased female—but not male—preference for *c*VA-rich food; and (*v*) microbiota-derived compounds only affected male upwind flight latency. Therefore, sex pheromones interact with food volatile chemicals to induce sex-specific flight responses in Drosophila.

## Introduction

When searching for food sources, prey, appropriate sexual partners, and the most suitable ecological niche, animals often use multiple sensory cues^1,2^. Chemical sensory stimuli are ubiquitously used by vertebrates, invertebrates and even unicellular organisms to fulfil these needs^3^. Very often, the simultaneous emission of food-derived molecules and pheromones helps individuals to accurately orient in space during their search^4–7^.

Similar to many other flying insects, Drosophila can fly in a sustained manner over a relatively long distance^8–11^. When flying insects are far from a food source, they generally use mechanosensory stimuli to estimate wind velocity, while they rely on chemical and visual stimuli to visually orient through an odour gradient^12–15^. At a closer distance, they simultaneously perceive visual and chemical signals before landing on the source^16–18^. To detect volatile chemosensory cues, *Drosophila melanogaster* flies use sensory hairs (sensilla) covering several head appendages (antenna, maxillary palps^19^) and the wings^20,21^. The receptor neurons housed in these sensilla send a converging influx to specific—sometimes sex-specific—brain centres that process the information to allow the insect to react with an adapted behaviour according to its sex and mating status^6,22–25^.

*D. melanogaster* flies mainly use two types of pheromones: 11-*cis*-vaccenyl acetate (*c*VA^26,27^), a male-specific volatile compound, and several sex-specific cuticular hydrocarbons (CHs), which are mostly—but not exclusively—detected by physical contact^28–31^. During copulation, males transfer *c*VA into the female reproductive tract; subsequently, the eggs laid on food are covered with *c*VA, but only during the few days following mating^32^. Drosophila pheromones can modulate several subsocial behaviours. For example, *c*VA associated with sex-specific CHs inhibits male courtship and stimulates female sexual receptivity^33–38^. The “male CHs + *c*VA” mixture can also induce male-male aggression^39,40^ and render food more attractive to both sexes^5,41^.

Moreover, some food-derived molecules (especially yeast and fermentation products) and pheromones are involved in Drosophila long- to close-distance attraction^21,42^. At a relatively close distance, fatty acid-derived compounds can influence aggregation and feeding^43,44^. Some of these products deposited in frass depend on the activity of gut-associated bacteria^43^. At a longer distance, *c*VA combined with volatile food by-products (vinegar) can increase fly aggregation on food sources^6,45,46^, depending on the sex, hunger state and mating status of the tested flies^5,47^. Furthermore, an aldehyde compound (Z4-11Al) derived from the degradation of the main cuticular pheromone (7,11-heptacosadiene) produced by females of most strains can attract wild-type males of the two sibling species *D. melanogaster* and *D. simulans* at some distance and with some intraspecific variation^48^. An inter- and intraspecific variation for free flight odour tracking has also been reported in several Drosophila species^49^.

To decipher the long-range effect of *c*VA and/or CHs combined with food-derived molecules, we measured free flight performance in *D. melanogaster* flies tested in a wind tunnel. Such devices have been used to test odour-guided flight in Drosophila in only a few studies^12,13,16,18,21,42,44,45,47,48^. Our experiments aimed to test the ecological significance of the chemical mixtures deposited by flies on food and not only the effect of isolated compounds. More precisely, compared to previous reports, the originality of our study resides in the combination of the six following aspects: we determined (*i*) both flight performance and food preference in individual flies (*ii*) of the two sexes (*iii*) from several natural and lab-manipulated lines (*iv*) presented with a dual food choice generally associating plain food with food labelled (*v*) by several lines of flies producing various CHs/*c*VA profiles (*vi*) or with an altered microbiota.

## Results

### Responses of control *D. melanogaster* flies to food labelled by other control flies

**Figure 1:**
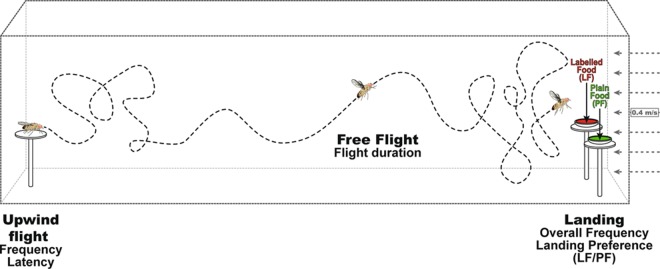
Wind tunnel device used to measure single fly responses to food choice. Schematic representation of upwind free flight in the wind tunnel. The four flight features measured are the frequency and latency of taking upwind flight, flight duration and overall landing frequency. We also noted the preference (LF/PF) in the binary choice between food generally consisting of plain food (PF) and food labelled by flies from different lines (labelled food = LF). The wind speed and direction are indicated on the right.

We measured several free flight parameters in individual flies: the frequency and latency of taking upwind flight from the starting platform, flight duration (corresponding to the time difference between “taking upwind flight” and “landing latency”) and the overall landing frequency (estimated only from individuals taking upwind flight; Fig. 1). Landing preference was measured in a dual-choice test based on the frequency of individuals landing on each food source. For the sake of clarity, we only show the upwind flight and overall landing frequencies together with the landing preference. The latency of taking upwind flight and the flight duration are shown in the Supplemental Info section.

**Figure 2:**
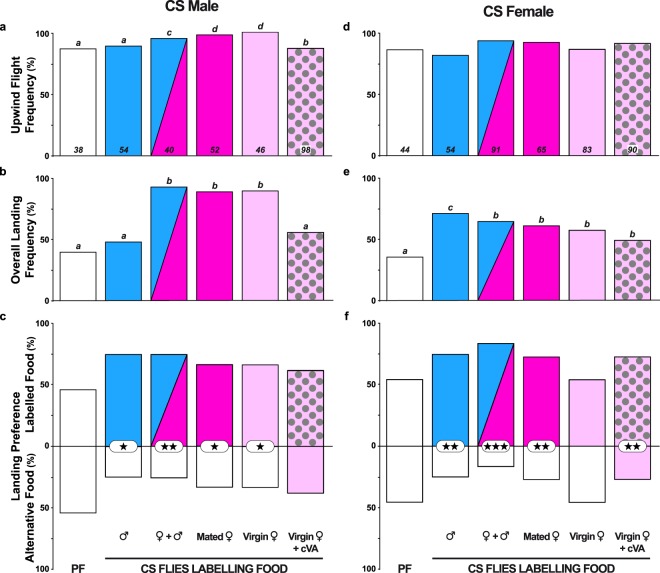
Flight and landing preference in single CS flies tested for food labelled by CS flies. The histograms represent (**a**,**d**) the frequency of flies taking upwind flight (calculated from the total number of flies tested: see the bottom of each histogram bar), (**b**,**e**) the overall landing frequency (calculated from flying individuals), and (**c**,**f**) the landing preferences of mature male (**c**) and female CS flies (**f**). The coloured fill of the bars indicates the sex and mating status of the CS flies labelling the food (labelled food = LF; indicated below the histograms). The blue and pink colours indicate food labelled by males and females, respectively. Light and dark pink colours indicate labelling by virgin and mated females, respectively. The mixed blue/dark pink histogram bar indicates food labelled by both sexes (mixed sex). The light pink bar with dark spots (right bar) indicates food labelled by virgin females and enriched with synthetic *cis*-vaccenyl acetate (*c*VA). The white bar on the left represents plain food (PF; without fly labelling). In all experiments, the flies were tested through a binary choice involving PF and LF, except in the control test (left bar: PF/PF) and the test involving “virgin CS female odours *vs*. virgin CS female odours + cVA” (right bars). Therefore, the bars above and below the baseline show the proportion of flies choosing each food type. For each sex, the difference between upwind flight and landing frequencies was tested with the Wilks *G*^2^ likelihood ratio test completed with a computation of significance by cell (Fisher’s exact test; significant differences at α = 0.05 are indicated by different letters above the bars), whereas landing preference was tested with the *z-test*, and the corresponding frequencies were compared between the different LFs using the Marascuilo procedure. For the two frequency parameters, significant differences are indicated by different letters at α = 0.05, while the level of significance for food preference is represented (or not) by asterisks (*α < 0.05; **α < 0.01; ***α < 0.001; no asterisk: not significant). (**a**) Wilks *G*^*2*^ likelihood ratio test, *G*^2^_(5df)_ = 16.6, *p* = 0.005; (**b**) *G*^2^_(5df)_ = 61.2, *p* < 10^−4^; (**c**) Marascuilo procedure*, Khi*^2^_(5df)_ = 4.4, *p* = 0.494; (**d**) *G*^2^_(5df)_ = 6.6, *p* = 0.249; (**e**) *G*^2^_(5df)_ = 15.3, *p* = 0.009; **(f**) *Khi*^2^_(5df)_ = 12.0, *p* = 0.035.

Upwind flight and landing preference were assessed in single starved control Canton-S (CS) male and female flies presented with a dual choice between plain food (PF) and fly-labelled food (LF; Fig. 2). All LFs were marked by groups of 170 CS flies (either males or females; or 85 flies of each sex in the “mixed sex odour” treatment). Note that while the control test did not involve LFs (“PF *vs*. PF”; see empty histogram bars on the left side of Fig. 2), another test simultaneously used two LFs (“CS virgin female odours” *vs*. “CS virgin female odours + *c*VA”; see histogram bars with dark dots on the right side).

CS males and females very frequently took upwind flight in the presence of all fly-labelled foods (85–100%; Fig. 2a,d). Only males showed significant differences, presenting higher frequencies of taking upwind flight toward food labelled by females. Both sexes showed significant variation in their overall landing frequencies depending on the genotype of the flies that labelled the food. Males landed more frequently (88–92%) on food with CS female odours (either mixed sexes, mated flies or virgins) than on foods with “CS virgin female odours + *c*VA” or with CS male odours (55 and 48%, respectively; Fig. 2b) and on PF in the choice test without fly-labelled food (PF/PF; 39%). Females generally landed more frequently on food with CS male odours (70%) than on any other fly-labelled food, while the PF/PF choice induced much less landing (34%, Fig. 2e). No marked difference was detected between the sexes and LFs for the latency of taking upwind flight or the flight duration (Fig. S1).

Landing preference significantly varied depending on both the sex of the fly and the type of food tested (Fig. 2c,f). Males preferred food with CS mix sex odours (*p* < 0.01; Fig. 2c) than food labelled with CS male or mated or virgin female odours (*p* < 0.05). However, males showed no landing preference toward food with CS virgin female odours enriched with *c*VA compared to CS virgin female odours without *c*VA (*p* > 0.05). Females strongly preferred food with CS mixed sex odours (*p* < 0.001; Fig. 2f) over food with CS male or mated female odours (*p* < 0.01). However, in contrast to males, females showed no landing preference toward food with CS virgin female odours (*p* > 0.05), while they clearly preferred food labelled with “CS virgin female odours + *c*VA” (*p* < 0.01).

To check whether the landing preference observed in the wind tunnel could also be observed with another behavioural device, we measured individual fly preference in a Y-maze olfactometer. In addition to the “PF *vs*. PF” choice (control test), we tested fly preference in the choice between PF and food with CS mixed sex odours (Fig. S2). In the latter choice test, males showed a faster and stronger response than females: after 20 min, 50% of males and 25% of females had moved to either arm (Fig. S2c); after 240 min, 75% of males and 70% of females had moved in either olfactometer arm, and they showed a clear preference toward food with CS mixed sex odours (*p* < 0.001 and *p* < 0.01, respectively; Fig. S2a,b).

### Responses of control *D. melanogaster* flies to food labelled by flies showing diverse cuticular hydrocarbon and cVA profiles

**Figure 3:**
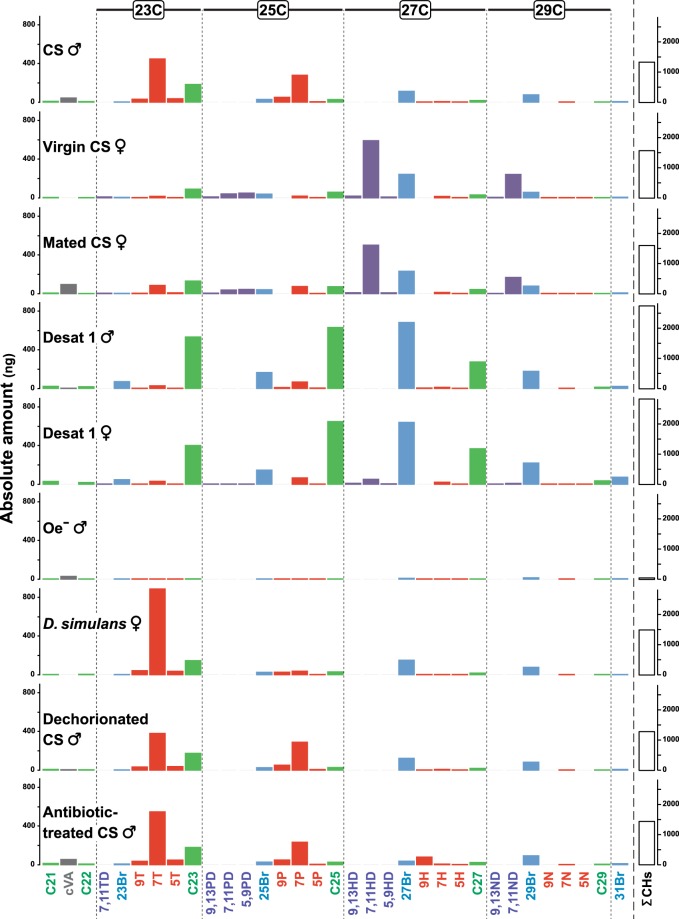
Chemical profiles of the flies labelling the food. For each fly genotype (indicated on the left), the mean absolute contents (in ng) of the principal cuticular hydrocarbons (CHs) detected and *c*VA are shown. For the sake of clarity, CHs were separated based on their chain length (indicated above the histograms for 23 to 29 carbons (C23-C29, and below histogram bars on the left for the lighter CHs with 21 and 22 carbons (C21, C22). The sum of CHs (∑CHs; in ng) is represented with unfilled bars on the right. *N* = 20 flies except for the Desat1 males and females, where *N* = 15. For the nomenclature of chemicals and fly line identity, please refer to Table S1 and Fig. 4.

To further determine the nature of the fly compounds potentially influencing the flight response, food labelled by flies from several lines differing in their cuticular hydrocarbon (CH) profiles or *c*VA levels, or with a potentially altered microbiota was used (Fig. 3; Tables S1 and S2).

**Figure 4:**
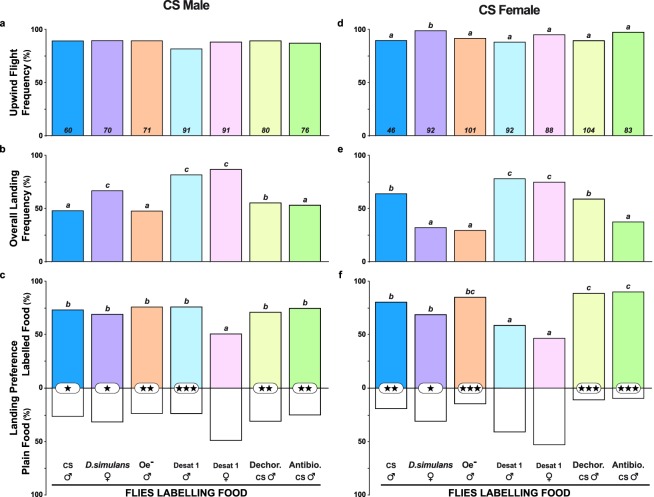
Free flight and landing preference in single CS flies toward food labelled by flies of various genotypes. The histograms represent (**a**,**d**) the frequency of flies taking upwind flight, (**b**,**e**) the overall landing frequency, and (**c**,**f**) the landing preference in mature male (**a**–**c**) and female CS flies. (**d**–**f**) The fly-labelled foods are shown below the histograms, from left to right: CS males, *D. simulans* females, oenocyteless males (Oe^−^), Desat1 mutant males and females, CS males originating from dechorionated eggs (Dechor. CS males) and antibiotic-treated eggs (Antibio. CS males). For the parameters and statistics, please refer to the Fig. 2 legend. (**a**) *G*^2^_(6df)_ = 3.2, *p* = 0.789; (**b**) *G*^2^_(6df)_ = 48.5, *p* = 0.0001; (**c**) *Khi*^2^_(6df)_ = 13.9, *p* = 0.031; (**d**) *G*^2^_(6df)_ = 13.1, *p* = 0.042; (**e**) *G*^2^_(6df)_ = 85.3, *p* = 0.0001; (**c**) *Khi*^2^_(6df)_ = 39.4, *p* = 0.0001. The sample size is indicated at the bottom of each histogram bar (**a**,**d**).

In these conditions, most flies took upwind flight (81–98%; Fig. 4a,d). Females showed a higher upwind flight frequency in the presence of food labelled with *D. simulans* female odours than related to any other fly-labelled food. *D. simulans* virgin females were used because they produce similar CHs to CS males but no *c*VA^28^. Food labelled with *D. simulans* female odours induced 60% and 36% overall landing frequencies in males and females, respectively; this food was preferred by both sexes (*p* < 0.05; Fig. 4c,f). Food labelled by oenocyteless males (Oe^−^; producing very low CH levels but wild-type-like *c*VA levels^50^) induced low overall landing frequencies in males and females (48% and 29%, respectively; Fig. 4b,d) but a strong landing preference (*p* < 0.01 and *p* < 0.001, respectively; Fig. 4c,f). In contrast, food labelled by *desat1* mutant males and females (producing low levels of desaturated CHs and high levels of linear saturated CHs^51,52^) induced high overall landing frequencies in both sexes (75 to 86%), but a significant landing preference was only shown by CS males toward food with *desat1* male odours (*p* < 0.001).

To test the possible effect of the microbiota potentially transferred by the flies labelling the food, we treated the eggs from which the “labelling” flies were produced. Practically, we either removed the egg chorion or raised control eggs on antibiotic-rich food^53,54^. While the two foods labelled by dechorionated- or antibiotic-treated CS males induced low to moderate overall landing frequencies (38–59%), both were strongly preferred by males (*p* < 0.01) and females (*p* < 0.001). No difference was detected for upwind flight latency, and only the female flight duration toward food with Oe^−^ odours was slightly shorter compared to the responses to other labelled foods (Fig. S3).

### Testing the relationship between fly chemicals, flight parameters and food preference

**Figure 5:**
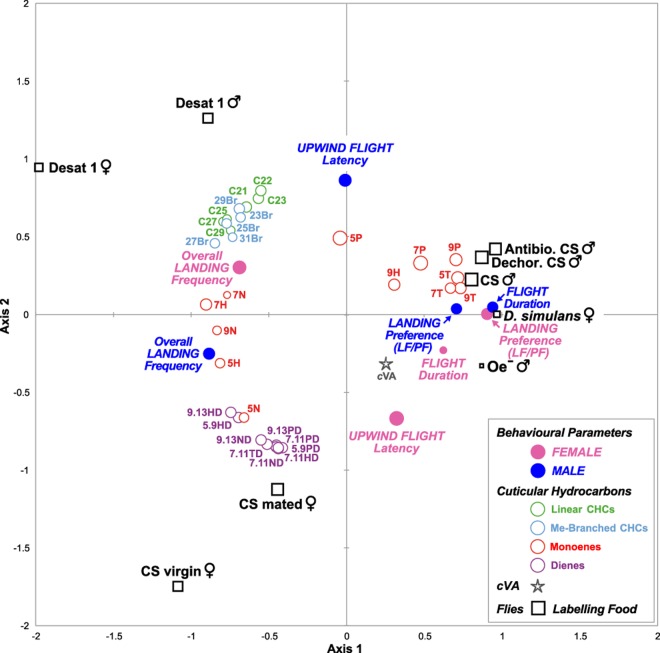
Statistical relationship between chemicals, flight parameters and labelling flies. Three-dimensional plot of the 3 first axes of the principal component analysis (PCA; type Pearson’s correlation matrix; with standardized values) using relevant flight parameters (upwind flight latency, flight duration, overall landing frequency), landing preference (labelled food *vs*. plain food = LF/PF) and the relative proportions of each hydrocarbon and *c*VA detected in the cuticular extract of labelling flies as variables and the “labelling” fly types as individuals. The 3^rd^ dimension (axis 3) is represented by the size of the symbols (circle, square, star). These three first PCA axes explain 84.5% of the total variation of our sample (axis 1 = 43.2%, axis 2 = 31.4%, axis 3 = 9.9%). Blue and pink filled circles represent male and female flight parameters, respectively. The star represents *c*VA, while empty circles represent the content of CHs carried by the labelling flies (red = monoenes; purple = dienes; blue = methyl-branched CHs; green = linear CHs). The fly lines used to label food are represented by empty black squares.

In an attempt to determine the roles of *c*VA, CHs, food and/or their derived products in affecting upwind flight and landing preference, we performed a principal component analysis (PCA) taking into account the variation of all relevant flight parameters (upwind flight latency, flight duration, overall landing frequency) and landing preference (“LF/PF”), together with the relative proportions of each hydrocarbon and *c*VA detected in the cuticular extracts of the flies used to label food (Fig. 3; Table S2). All these parameters were used as variables, whereas the “labelling” fly types were considered as individuals (Fig. 5). The three first axes of this PCA explained 84.5% of the total variation of our sample (170 flies for CH analysis; 769 males and 943 females for behaviour). Most of the flight parameters were highly correlated between the sexes. In both sexes, the overall landing frequency was strongly negatively correlated with the (*i*) labelled food preference and (*ii*) flight duration. In other words, when the total number of flies landing on both food sources increased, their preference for landing on the labelled food decreased, and their flight duration increased. Although we cannot infer the effect of each single chemical in this PCA given that multiple chemicals often act in synergy to modulate one or several aspects of flight, we noted several clear correlations between behavioural parameters and (groups of) chemical compounds. More precisely, in both sexes, the landing preference toward labelled food was highly correlated with the labelling of the preferred food by flies producing high levels of shorter-chain male-predominant monodesaturated CHs (monoenes with 23C-carbon chains: 5 T, 7 T, 9 T; with 25C-carbon chains: 7 P, 9 P). In contrast, the overall landing frequency of males and females showed a positive relationship with the level of longer-chain monoenes (with 27 C: 5 H, 7 H; with 29 C: 7 N, 9 N) on the labelled food. Increased overall landing frequencies were also marginally correlated with female-specific dienes (double desaturated CHs) in males and with methyl-branched (23Br-31Br) plus linear saturated CHs (C21-C29) in females. Note that the upwind flight latency showed a negative relationship between the sexes. As a consequence, the female latency of taking upwind flight and, to a lesser extent, the female flight duration were correlated with the variation of the *c*VA level, which were effects that were not detected in males.

### Flight and food preference in naïve male flies of diverse genotypes

**Figure 6:**
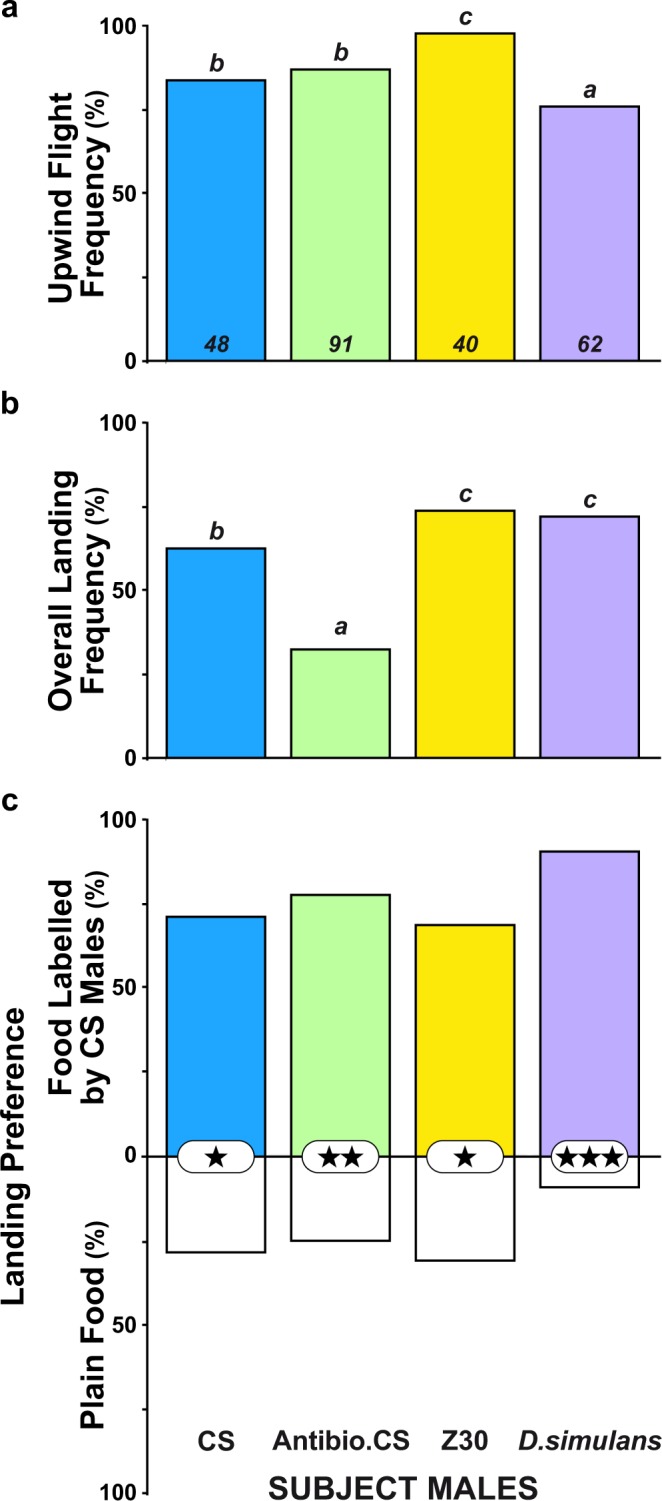
Free flight and landing preference in single male flies from various genotypes toward food labelled by CS males. Histograms represent (**a**) the frequency of flies taking upwind flight, (**b**) the overall landing frequency, and (**c**) the landing preference between plain food (PF; empty bars) and food labelled by CS males (coloured bars). In addition to CS control males (blue coloured bars), we tested the response of individual males derived from CS antibiotic-treated eggs (Antibio. CS males; green coloured bars), from the Zimbabwe30 wild-type line (Z30; yellow coloured bars), and from *D. simulans* (*D. simulans*; purple coloured bars). For parameters and statistics, please refer to the Fig. 2 legend. (**a**) *G*^2^_(3df)_ = 11.0, *p* = 0.012; (**b**) *G*^2^_(3df)_ = 7.9, *p* = 0.049; (**c**) *Khi*^2^_(3df)_ = 5.5, *p* = 0.142. The sample size is indicated at the bottom of each histogram bar (**a**).

We compared the response shown by control CS males toward food labelled with CS male odours with the response of males from (*i*) an antibiotic-treated CS strain, (*ii*) another *D. melanogaster* wild-type strain producing variant CHs (Zimbabwe30 = Z30^55^), or (*iii*) the *D. simulans* sibling species (Fig. 6). These males showed significant variation in their upwind flight frequencies (76% in *D. simulans*, 98% in Z30 males) and landing frequencies (approximately 74% in Z30 and *D. simulans*; 34% in antibiotic-treated males). Both the latency of taking upwind flight and flight duration varied strongly between males (*p* < 0.001; Fig. S4). More particularly, antibiotic-treated CS males showed a shorter upwind flight latency compared to the three wild-type males (*p* < 0.001). Moreover, *D. simulans* and antibiotic-treated CS males showed a greater landing preference toward food labelled with CS male odours (*p* < 0.001 and *p* < 0.01, respectively) compared to those of the two other males (*p* < 0.05).

## Material and Methods

### Drosophila strains and rearing

We used the following strains: two *Drosophila melanogaster* wild-type strains: Canton-S (CS) and Zimbabwe30 (Z30), a *Drosophila simulans* wild-type strain (line #K509, a gift from Prof. Yamamoto), and homozygous *desat1*^1573^ mutant flies, showing altered production and perception of cuticular hydrocarbons^51,52,56^. We also used oenocyteless flies (Oe^−^^50^) resulting from a cross between UAS-*hid*/*CyO* females and +: PromE(800)-Gal4[2 M],Tub:Gal80^ts^, + males (kindly provided by Prof. J-C Billeter). All strains were raised on yeast/cornmeal/agar medium [for 6 L of food: 300 g of yeast, 392 g of maize flour, 55 g of agar and 185 ml of Tegosept (®Apex) completed with distilled water] and kept under a 12:12 hour light/dark cycle at 24 ± 0.5 °C with 65 ± 5% humidity. Oe^−^ transgenic parents were mated and kept at 18.5 °C until the emergence of their adult progeny, which were kept at 29 °C for 3–5 days.

All flies were derived from mass-rearing stocks transferred every 2–3 days to avoid competition and regularly provide progeny. The flies were screened 4 to 6 hours after emergence under light CO_2_ anaesthesia and kept at 24 ± 0.5 °C (at 29 °C for Oe^−^ flies) in same sex groups (20 flies) in all labelling flies and the female fly subjects. Male subjects were held individually to prevent social interactions potentially affecting behaviour^57^.

### Food labelling

To investigate the effect of the molecules potentially involved in free flight odour tracking and landing preference, we labelled fresh plain laboratory food with living flies. To label food, 170 “labelling” flies were kept in a petri dish (∅ = 5.4 cm) filled with fresh food and covered by a plastic lid (h = 6.4 cm) for 24 hours under similar experimental conditions as described above. Flies used to label food (“labelling flies”) were removed prior to the behavioural experiment.

### Microbiota alteration

Three- to four-day-old mass-reared CS females were allowed to lay eggs for 6 hours in an egg-laying device consisting of a 50 mm Petri dish filled with 1 ml of 3% agar striped with fresh yeast to stimulate egg laying. After this period, the flies were removed, and their eggs were collected. To remove the chorion associated with the microbiota present on the surface, eggs were soaked for a few seconds in concentrated -bleach water (9.6%). These eggs were then thoroughly rinsed with distilled water and deposited in groups of approximately 130 in vials with fresh food. For the antibiotic treatment, a dozen of 4-day-old flies of each sex were kept together overnight for mass mating. The next morning, males and females were separated under light CO_2_ anaesthesia. Then, females were allowed to lay eggs on antibiotic-treated food (50 μg/mL tetracycline +200 μg/mL rifampicin +100 μg/mL streptomycin) during a 12-hour period^53^. Adult males resulting from these antibiotic-treated eggs were used to label food under similar conditions to those described above.

### Wind tunnel

The design of the wind tunnel has been previously described in detail^21,49,58^. Briefly, it consists of clear acrylic (length = 155 cm; width and height = 30.5 cm) and is illuminated by four band strips of white LEDs (BDL- F300W-05-3528, Boulevard des Leds, France; length = 1 m) located below the tunnel base and separated with a red screen. Tracing paper was placed over the tunnel to homogenize the light intensity inside the flying section. The two lateral panels of the tunnel were covered with a randomized pattern consisting of black and white squares (side = 3 cm). A “departure/starting” platform (height = 16 cm) was placed in the downwind section at 90 cm from the two landing platforms (height = 16 cm, ∅ = 1.7 cm) located in the upwind section. The two landing platforms were placed 10 cm from each lateral panel and were separated from each other by 7.5 cm. Before each behavioural test, approximately 1 cm^3^ of food was deposited on a microscope slide at the top of each platform. A humidifier (® OKOIA, AH400; Tianjin, China) was placed at the entrance of the airflow to maintain a constant humidity (65–75%) in the flying section. A laminar airflow (0.4 ms^−1^) was running through the section. After each session of tests (performed between 9:00 am and 3:00 pm), the wind tunnel was washed with a 70% ethanol solution, and the room was ventilated until the next day. One hour before the start of the experiment, the room temperature was adjusted to 24±1 °C.

### Free flight parameters and food choice

The experiments were conducted under red light with starving flies to stimulate upwind flight attraction^46^. More precisely, 16 hours before the test, each fly was placed in a glass vial containing only a piece of absorbent paper (length = 4 cm, width = 2 cm) moistened with 90 μL of distilled water and kept at 25 °C. During flight, we measured several parameters and landing preference in a binary food choice assay. Individual flies were introduced with a mouth aspirator to an acclimation chamber (consisting of an acrylic tube; ∅ = 5 mm) separated by a gate from the inside of the wind tunnel and were left for 3 min. After acclimation, the flies were allowed to reach the part of the tube opening inside the wind tunnel. Once the fly reached the lift off platform, we successively noted its latency (and frequency) of taking upwind flight, flight duration (between lift off and landing on either platform, also corresponding to landing latency), and in the case of landing, the platform chosen. We also determined the overall landing (based on the sum of landings on the two platforms). Each experiment lasted 10 min or less when the fly landed on a platform. Each binary choice experiment generally associated one platform with plain food and the second one with fly-labelled food. In the case of food labelled by virgin females and enriched with *c*VA, 100 ng of *c*VA (® Cayman Chemical, Ann Arbor, MI, USA; 50 mg/ml solution in ethanol; purity >98%) in 5 μl of hexane was added to a Whatman filter paper patch (∅ = 1 cm, ® GE Healthcare Life Sciences) and deposited on fly-labelled food a few minutes prior to each test. In this test, 5 μl of hexane was added to filter paper on food labelled by virgin females (without *c*VA) on the “control” platform.

### Y-maze olfactometer

Food preference was also measured in a glass Y-maze, which allowed us to record the walking decisions made by individual flies confronted with two odours present in either arm of the maze. The device consisted of a regular empty glass vial connected to a straight tube (length = 6 cm; ∅ = 0.3 cm) divided into two 5-cm long arms, each of which was connected to a glass vial containing a filter paper impregnated with the food to be tested. The Y-maze was placed in darkness, and the tests were performed between 9 am and 2 pm. The flies and fly-labelled food were prepared as for the wind tunnel experiment. Each fly was individually introduced into the starting vial connected to the straight arm of the Y-shape olfactometer. Every 20 min, after the start of the test (and during the first two hours of the test), the position of the fly was noted in the device relative to the arm (type of food) chosen. The final choice of the fly was also noted 240 min after the start of the test.

### Chemical analysis

To extract adult cuticular hydrocarbons, 5-day-old flies were individually immersed for 10 min at room temperature in 30 μl of hexane containing 100 ng of C26 (n-hexacosane) and 100 ng of C30 (n-triacontane) as internal standards. Each fly was removed, and the extracts were stored at −18 °C until analysis. All extracts were analysed using a Varian CP3380 gas chromatograph fitted with a flame ionization detector, a CP Sil 5CB column (25 m × 0.25 mm internal ∅; 0.1 μm film thickness; Agilent), and a split–splitless injector (60 ml/min split-flow; valve opening 30 s after injection) with helium as the carrier gas (velocity = 50 cm/s at 120 °C). The temperature program began at 120 °C, was then ramped at 10 °C/min up to 140 °C, followed by 2 °C/min to 290 °C and holding for 10 min. The chemical identities of the major cuticular hydrocarbons and *c*VA were checked using gas chromatography-mass spectrometry^30^. The amount (in ng) of each compound was calculated based on the values provided by internal standards.

### Statistics

Behavioural frequencies (upwind flight and landing) were compared using the Wilks *G*^2^ likelihood ratio test completed with a computation of significance by cell (Fisher’s exact test). The choice between the two platforms was tested using the *z-test*, and the corresponding frequencies were compared between the different fly-labelled foods using the Marascuilo procedure. We performed a principal component analysis (PCA; Pearson’s correlation matrix type; with standardized values) with the relevant flight parameters (upwind flight latency, flight duration, overall landing frequency), food preference (LF/PF) and the level of each hydrocarbon and of *c*VA detected in the cuticular extracts of the “labelling flies” taken as variables, while the “labelling fly” strains were used as individuals. PCA is a powerful type of analysis that has often been used in similar studies to discover correlations between individual chemical compounds present in complex mixtures and behavioural events potentially induced by these compounds^59–66^. Finally, the dynamic curves obtained for the latency of upwind flight and flight duration (shown in Supplemental Figures) were analysed using the Kaplan-Meier procedure followed by a log-rank test and a post hoc multiple pairs comparison (α = 0.05 with Bonferroni correction). All statistical analyses were performed using XLSTAT 2019^67^.

## Discussion

Our study aimed to investigate the potential effect of CHs and *c*VA mixed with laboratory food on the long-range olfactory tracking behaviour of Drosophila flies. Compared to other Drosophila studies performed in a wind tunnel, the originality of our approach lies in the binary choice between two food sources, allowing us to determine the landing preference after upwind flight. The wide array of chemical profiles shown by the flies used to label food was helpful in attempting to establish a relationship between pheromonal compounds and flight response. The comparison of the responses shown by the control flies of both sexes and by several variant males provided us with additional data. The dissociation between the “overall landing frequency” and “landing preference” provides novel information suggesting that the perception of some volatile olfactory cues—mostly non-specific—emanating from fly-labelled food can influence upwind flight and non-discriminatory landing, while other cues that are more specific and perceived at a closer distance to the food sources can influence landing preference and increase flight duration. Nevertheless, when testing chemical blends, one can never assume the influence played by each single component on each single behavioural parameter^68^. Another limitation is due to the fact that the concentration of the various chemicals participating to the blend can change between the source point to the fly position during its flight. Therefore, it is never easy to determine which component(s) of the blend is (are) active at every position of the fly in the wind tunnel. The different aspects of this hypothesis are discussed below.

### Taking upwind flight

The different volatile substances emanating from the fly-labelled food may not have been perceived by the control flies (CS) starting their upwind flight since they showed no—or very little—food-related difference in either the latency or the frequency of upwind flight. This was confirmed by the absence of a correlation between upwind flight latency and CHs in our PCA. However, the negative correlation between the sexes for the latency of taking upwind flight indicates sex-specific variation relative to the perception of *c*VA when flies decide to take upwind flight. Three more observations made in the present study reinforce this hypothesis: (*i*) both the female latency of taking flight and female flight duration were moderately correlated with *c*VA amounts; (*ii*) females—but not males—preferred to land on food labelled with virgin CS female odours + *c*VA compared to food labelled with virgin CS female odours without *c*VA; and, (*iii*) food with mixed sex odours (described as copulating fly^46^ or mated^5^ odour) was slightly preferred by control females compared to control males. Together, these data support the finding that starved females exhibit a higher sensitivity than males to *c*VA associated with food-derived odour(s)^5^. However, the stronger preference shown by males toward food with mixed sex odours in the Y-olfactometer suggested that the sex difference observed in the wind tunnel disappeared when fly odours were more concentrated in a smaller-volume behavioural device without wind or when flies walked instead of flying.

In contrast to previous reports, the control males did not show any long-range attraction to food that was either naturally or experimentally labelled with *c*VA^45^. At least five experimental factors could explain the discrepancies between our study and other reports: (*i*) we tested single flies, while others tested groups of flies (between 10^46^ and 1000^45^), and we suspect that in groups, the first flies contacting the source could emit additional pheromones that subsequently affect the behaviour of other flies; (*ii*) we tested food choice in a wind tunnel, in contrast to the two previous studies that used an olfactometer placed inside a wind tunnel; (iii) we tested food labelled by flies but not fly extracts^45^ or intact flies kept behind a mesh^46^; (*iv*) in other studies, *c*VA was not mixed with food but was presented concurrently with vinegar, a food-derived compound that strongly reinforces the *c*VA effect^6,46^; and (*v*) the sex-specific response to *c*VA may also be due to the sex-specific effect of *c*VA conditioning during early developmental exposure^32^.

In addition to *c*VA, long-range attraction to male odours or copulating fly odours could also be induced by some as-yet-unknown volatile male substance(s) emitted just prior to copulation^46,69,70^. This possibility was not clear in our hands given that the control flies only showed a slightly increased preference toward food labelled with CS mixed sex odours compared to food with CS male odours. Both CS sexes also showed a strong preference toward landing on food labelled by CS males with an altered microbiota. Given that such “labelling” males (either originating from dechorionation or antibiotic treatment) showed no CH defect compared to control males (Fig. 3 and in contrast with a study involving antibiotic-treated flies^53^), it is possible that the hypothetical compounds that are normally derived from fly microbiota activity tend to partially repel fly landing. Compared to previous studies revealing the strong effect of frass deposited both by control and bacterially infested flies^43,71^, our results were surprising given that the food on which 170 flies were kept during 24 hours may be rich in frass. However, the apparent discrepancy between studies could again be related to the very different sizes of the experimental devices used to test fly behaviour.

The Z30 and CS antibiotic-treated males diverged from the control males in several flight parameters in the presence of food labelled with CS male odours. The flight duration of Z30 males was longer compared to those of other males, but this did not affect their overall landing frequency, which was still higher than that of CS males. The variation of Z30 behaviour could be linked to the lower activity of these flies in the presence of plain food^49^ and more generally to their lower behavioural activity^72^. In contrast, CS antibiotic-treated males took flight more quickly, which could have resulted from either their altered olfactory perception, as shown in germfree mice^73^, or the lack of exposure to microbiota-derived pheromones during their early development^54^.

### Landing on the food source

The control flies approaching food/pheromonal sources spent a greater proportion of time there, likely due to more intense sampling of cues in the surroundings these sources^49^. Then, based on the simultaneous integration and comparison of the visual and chemical stimuli emanating from each platform (in the case of a binary choice), the fly chooses to land on one of the platforms^18^. We found that a higher preference for landing on fly-labelled food was correlated with a longer flight duration. This finding supports the idea that this decision-making process takes more time than non-discriminatory landing. This effect may also explain the shorter flight duration shown by *desat1* mutant males (with altered sex pheromone perception), especially near a food source^21,49,52^.

The difference between the low overall landing frequency induced by the control test without fly-labelled food (PF/PF; 34% in females, 39% in males) and the higher frequency (49–92%) induced by most experiments involving at least one type of fly-labelled food provides crucial information indicating that the compounds left by flies on food generally increase the overall landing frequency and never decrease it. Reciprocal sex-related effects were observed: the male landing frequency increased on food labelled by CS females, whereas the CS female landing frequency slightly increased on food labelled by CS males. Moreover, the food labelled with CS mixed sex odours induced the greatest landing preference in both sexes. This finding is in accord with a report showing male preference for mixed sex odours (added with vinegar in the physical absence of flies^46^). However, the last study did not report on female preference.

The high correlation shown by our PCA indicates that longer-chain—mostly non-sex-specific— CHs promote fast and frequent but non-discriminatory landing overall in both sexes (5-, 7- and 9-monenes—mono-desaturated CHs—with 27 and 29 carbons) or in females (saturated linear CHs, methyl-branched CHs). Once taken into account the limitation to evaluate the role that single components of a blend can play on distinct flight parameters, we propose the following hypothesis. At a longer distance (corresponding to the 80 cm of our wind tunnel), attraction (but no landing preference) could be induced by chemicals derived from these long chain CHs, as shown for (*Z*)−4-undecenal (*Z*4-11Al), derived from 7,11-heptacosadiene, in a no-choice experiment^46^. However, when the CS flies had the choice, they preferred to land on the food labelled with shorter-chain CHs (monoenes with 23 and 25 carbons) than with longer-chain CHs (and/or with saturated linear CHs). Given the wind velocity and the relatively low volatility of these shorter-chain monoenes^31^, they may be only perceived at a relatively short distance, just before landing. In addition to CH-derived products, food-derived molecules resulting in microbiota activity could also be involved in landing preference^43,71^. However, our study does not support this hypothesis given that food labelled by microbiota-treated flies induced a greater landing preference compared to food labelled by control (untreated) male flies.

In summary, our data indicate that odour-guided flight and landing preference toward a food source that is either marked with fly substances or not depend on both the chemicals left on the food by flies and the sex or strain of the flying insect. The behavioural response induced by such chemicals mixed in the food can vary when they are presented alone or simultaneously with a second food source.

## Supplementary information

Supplemental information

Supplemental information

## Data Availability

A xlsx File containing all raw data is available as supplemental material.
