# Supplementary material for: Flying Drosophila show sex-specific attraction to fly-labelled food
Source: Sci Rep. 2019 Oct 18;9:14947. doi: 10.1038/s41598-019-51351-1 (PMC6802089; doi:10.1038/s41598-019-51351-1)
Supplement: Supplementary file 1 — Supplemental information [file 41598_2019_51351_MOESM1_ESM.pdf]

Flying Drosophila show sex-specific attraction to fly-labelled food

Laurie Cazalé-Debat[1], Benjamin Houot[1,2], Jean-Pierre Farine[1], Claude Everaerts[1] and Jean-François Ferveur[1*]

[1] Centre des Sciences du Goût et de l'Alimentation, UMR6265 CNRS, UMR1324 INRA, Université de Bourgogne Franche-Comté, 6, Bd Gabriel, 21000 Dijon, France

[2] Current adress: Laboratoire de Biologie Fonctionnelle et Adaptative, UMR8251, Université Paris Diderot - Paris 7 / CNRS Equipe Processus Dégénératifs, Stress et Vieillissement Bâtiment Buffon, 4, rue Marie Andrée Lagroua Weill Halle 75013 Paris, France

SUPPLEMENTAL MATERIAL

(Tables S1 & S2; Figures S1-S4)

Table S1: Nomemclature of the principal cuticular hydrocarbons and *c*VA detected on the fly cuticule. (The color code corresponds to that shown in Figure 3).

Abbreviation	Chemical name
**C21**	n-Heneicosane
***c*VA**	(*Z*)-11-Vaccenyl acetate
**C22**	n-Docosane
**7.11TD**	(*Z,Z*)-7,11-Tricosadiene
**23Br**	2-Methyldocosane
**9T**	(*Z*)-9-Tricosene
**7T**	(*Z*)-7-Tricosene
**5T**	(*Z*)-5-Tricosene
**C23**	n-Tricosane
**9,13PD**	(*Z,Z*)-9,13-Pentacosadiene
**7,11PD**	(*Z,Z*)-7,11-Pentacosadiene
**5,9PD**	(*Z,Z*)-5,9-Pentacosadiene
**25Br**	2-Methyltetracosane
**7P**	(*Z*)-7-Pentacosene
**5P**	(*Z*)-5-Pentacosene
**C25**	n-Pentacosane
**9,13HD**	(*Z,Z*)-9,13-Heptacosadiene
**7,11HD**	(*Z,Z*)-7,11-Heptacosadiene
**5,9HD**	(*Z,Z*)-5.9-Heptacosadiene
**27Br**	2-Methylhexacosane
**7H**	(*Z*)-7-Heptacosene
**5H**	(*Z*)-5-Heptacosene
**C27**	n-Heptacosane
**9,13ND**	(*Z,Z*)-9,13-Nonacosadiene
**7,11ND**	(*Z,Z*)-7,11-Nonacosadiene
**29Br**	2-Methyloctacosane
**9N**	(*Z*)-9-Nonacosene
**7N**	(*Z*)-7-Nonacosene
**5N**	(*Z*)-5-Nonacosene
**C29**	n-Nonacosane
**31Br**	2-Methyltriacontane

Table S2: Production of the principal cuticular hydrocarbons and *c*VA in individual flies of all lines used to label food (mean ± sem).

MALES	Cs M	Antibiot. Cs M	Dechorion. Cs M	Desat 1 M	Z30 M	Oe- M	*D. simulans* M
**C21**	11 ± 1	16 ± 1	9 ± 1	24 ± 1	1 ± 0		4 ± 0
***c*VA**	45 ± 10	56 ± 11	6 ± 1	1 ± 0	23 ± 4	107 ± 66	95 ± 20
**C22**	8 ± 0	10 ± 0	6 ± 0	20 ± 1	1 ± 0		9 ± 1
**23 Br**	1 ± 0	9 ± 0	1 ± 0	72 ± 2	1 ± 0		1 ± 0
**9T**	33 ± 3	37 ± 2	34 ± 2	1 ± 0	12 ± 1		57 ± 3
**7T**	449 ± 21	549 ± 12	381 ± 15	29 ± 2	152 ± 6	2 ± 0	714 ± 30
**5T**	38 ± 1	50 ± 1	38 ± 1	4 ± 0	107 ± 5		40 ± 2
**C23**	186 ± 6	180 ± 5	176 ± 5	535 ± 12	42 ± 1	1 ± 0	169 ± 8
**25Br**	30 ± 3	29 ± 2	26 ± 1	41 ± 1	25 ± 2	1 ± 0	29 ± 2
**9P**	53 ± 3	51 ± 3	52 ± 1	167 ± 5	49 ± 3	1 ± 0	25 ± 4
**7P**	280 ± 16	235 ± 11	290 ± 11	11 ± 1	146 ± 8	1 ± 0	61 ± 3
**5P**	6 ± 1	6 ± 1	8 ± 1	69 ± 2	17 ± 1		1 ± 0
**C25**	30 ± 1	27 ± 1	29 ± 1	7 ± 1	20 ± 1	1 ± 0	34 ± 2
**27 Br**	114 ± 5	38 ± 1	124 ± 3	633 ± 9	82 ± 6	7 ± 0	153 ± 5
**9H**	0 ± 0	81 ± 2	0 ± 0	685 ± 21	1 ± 0	1 ± 0	0 ± 0
**7H**	8 ± 1	8 ± 1	9 ± 0	14 ± 1	6 ± 1	1 ± 0	1 ± 0
**5H**	0 ± 0	0 ± 0	0 ± 0	276 ± 6	1 ± 0		0 ± 0
**C27**	19 ± 1	25 ± 1	19 ± 1	7 ± 0	16 ± 1	1 ± 0	14 ± 2
**29 Br**	78 ± 3	94 ± 3	83 ± 3	8 ± 1	71 ± 4	18 ± 1	66 ± 7
**7N**	0 ± 0	0 ± 0	0 ± 0	180 ± 5	1 ± 0	2 ± 0	2 ± 1
**C29**	3 ± 0	7 ± 0	2 ± 0	14 ± 0	4 ± 0	1 ± 0	2 ± 0
**31Br**	7 ± 0	12 ± 1	9 ± 1	23 ± 1	10 ± 1	8 ± 0	4 ± 0
**C31**				2 ± 1			
**∑ HCs**	1354 ± 46	1466 ± 21	1296 ± 25	2823 ± 36	763 ± 21	46 ± 2	1387 ± 46
**∑ Desaturated**	867 ± 31	1018 ± 19	813 ± 20	135 ± 4	490 ± 13	7 ± 1	902 ± 34
**∑ Ramified**	231 ± 10	183 ± 4	242 ± 6	1135 ± 25	189 ± 10	34 ± 1	252 ± 10
**∑ Linear**	256 ± 8	265 ± 6	241 ± 6	1533 ± 21	84 ± 2	5 ± 0	232 ± 7

FEMALES	Cs F	Antibiot. Cs F	Dechorio. Cs F	Mated Cs F	Desat 1 F	*D. simulans* F
**C21**	5 ± 0	2 ± 0	6 ± 1	8 ± 1	31 ± 1	4 ± 0
*c*VA				96 ± 15		
**C22**	3 ± 0	4 ± 0	3 ± 0	4 ± 0	19 ± 1	6 ± 0
**7.11TD**	11 ± 0	10 ± 1	7 ± 0	6 ± 0	0 ± 0	
**23Br**	7 ± 0	5 ± 0	4 ± 0	5 ± 1	48 ± 1	1 ± 0
**9T**	4 ± 1	4 ± 0	3 ± 0	5 ± 0	1 ± 0	43 ± 1
**7T**	17 ± 1	17 ± 1	13 ± 1	86 ± 4	32 ± 1	889 ± 21
**5T**	2 ± 0	2 ± 0	1 ± 0	10 ± 1	4 ± 0	37 ± 1
**C23**	91 ± 2	108 ± 2	103 ± 5	132 ± 4	404 ± 9	148 ± 4
**C24**					22 ± 0	
**9,13PD**	11 ± 0	12 ± 1	9 ± 1	7 ± 0		
**7,11PD**	42 ± 1	40 ± 2	38 ± 2	39 ± 3	4 ± 0	
**5,9PD**	50 ± 2	35 ± 2	41 ± 2	45 ± 2	1 ± 0	
**25Br**	40 ± 1	46 ± 2	46 ± 2		147 ± 6	26 ± 1
**9P**				42 ± 2	11 ± 2	28 ± 2
**7P**	19 ± 1	21 ± 1	22 ± 2	75 ± 3	67 ± 3	38 ± 2
**5P**	2 ± 0	2 ± 0	2 ± 0	4 ± 0	4 ± 0	0 ± 0
**C25**	59 ± 2	65 ± 2	68 ± 3	73 ± 3	650 ± 15	31 ± 1
**9,13HD**	19 ± 1	21 ± 1	15 ± 1	11 ± 1		
**7,11HD**	593 ± 16	600 ± 11	577 ± 26	503 ± 21	49 ± 5	
**5,9HD**	10 ± 0	9 ± 0	9 ± 0	10 ± 0	6 ± 0	
**27Br**	244 ± 8	217 ± 4	206 ± 12	233 ± 8	642 ± 24	152 ± 4
**9H**						0 ± 0
**7H**	16 ± 2	33 ± 1	18 ± 2	14 ± 1	23 ± 1	0 ± 0
**5H**	2 ± 0	3 ± 0	1 ± 0	1 ± 0	2 ± 0	0 ± 0
**C27**	31 ± 2	50 ± 3	40 ± 2		369 ± 27	19 ± 1
**28Br**					9 ± 0	
**C28**				44 ± 2	9 ± 1	
**9,13ND**	7 ± 0	10 ± 1	6 ± 0	4 ± 0		
**7,11ND**	243 ± 9	285 ± 9	199 ± 12	169 ± 11	11 ± 1	
**29Br**	58 ± 2	83 ± 3	79 ± 3	79 ± 4	222 ± 6	78 ± 3
**9N**	0 ± 0	1 ± 0	0 ± 0	0 ± 0		
**7N**	1 ± 0	2 ± 0	1 ± 0	0 ± 0		0 ± 0
**5N**	0 ± 0	0 ± 0	0 ± 0	0 ± 0		
**C29**	3 ± 0	7 ± 0	5 ± 0	4 ± 0	37 ± 6	1 ± 0
**31Br**	7 ± 0	13 ± 1	14 ± 1	9 ± 1	74 ± 5	4 ± 0
**∑ HCs**	1599 ± 36	1708 ± 14	1537 ± 36	1624 ± 43	2901 ± 59	1505 ± 32
**∑ Desaturated**	1049 ± 23	1107 ± 13	963 ± 28	991 ± 29	216 ± 10	1035 ± 24
**∑ Ramified**	357 ± 10	365 ± 6	349 ± 14	369 ± 11	1142 ± 29	261 ± 5
**∑ Linear**	193 ± 5	237 ± 4	225 ± 8	265 ± 7	1542 ± 37	209 ± 6

SUPPLEMENTAL FIGURES

Figure S1: Cumulated frequency for the latency to to take upwind flight and for flight duration in control CS flies to food labelled by CS flies.

The cumulated frequencies represent the latency at which flies take upwind flight (top diagrams) and the flight duration (equivalent to landing latency; bottom) in CS males (left) and females (right). The data correspond to those shown on Figure 2. No statistical difference was detected between sexes and LFs. Curves were compared using Kaplan-Meier procedure with a subsequent Log-rank test. : (a) males upwind flight: $Khi^2_{(5df)} = 9.7$, $p= 0.08$, (b) females upwind flight : $Khi^2_{(5df)} = 5.7$, $p= 0.33$, (c) male overall landing = $Khi^2_{(5df)} = 3.3$, $p= 0.65$, (d) females overall landing $Khi^2_{(5df)} = 3$, $p= 0.70$

Figure S2: Labelled food preference in a Y-maze olfactometer.

The response of individual CS male (blue colour, left histograms and blue curve) and female flies (right bars and pink coloured bars and curve) was tested in a Y-maze olfactometer with a binary choice either consisting of two plain food (PF/PF, left choice for each sex) or PF with food labelled by mix sexes (LF; right choice). (**a**) Histograms represent the frequency of flies making a choice, after 240 min (the sample size is indicated at the base of each bar). (**b**) Representation of the food choice inside the Y-maze olfactometer. (**c**) Cumulated frequencies of flies responding during 240 min. The proportion of responding flies was tested with a Wilks G^2 likelihood ratio test completed with a computation of significance by cell (Fisher's exact test; significant differences at level $\alpha = 0.05$ are indicated by different letters above the bars). Food preference was tested with a *z*-test, and the corresponding choice frequencies were compared for both sexes and between the different choices using a Marascuilo procedure. The level of significance for preference is represented (or not) by stars (**: $\alpha < 0.01$; ***: $\alpha < 0.001$; no star: not significant). (**a**): $G^2_{(3df)}=22.7$, $p<10^{-4}$; (**b**): $Khi^2_{(5df)}=21.8$, $p<10^{-4}$.

Figure S3: Cumulated frequency for the latency to take upwind flight and for flight duration in control CS flies tested to food labelled by various genotypes.

The data correspond to those shown on Figure 4. Curves were analysed using Kaplan-Meier procedure with a subsequent Log-rank test. (a) Males upwind flight: $Khi^2_{(6df)} = 8.8$, $p = 0.18$, (b) females upwind flight: $Khi^2_{(6df)} = 10.5$, $p = 0.106$, (c) males overall landing $Khi^2_{(6df)} = 5.6$, $p = 0.47$, (d) females overall landing: $Khi^2_{(6df)} = 10.5$, $p = 0.104$. For more information, please refer to Fig. S1.

Figure S4: Cumulated frequency for the latency to take upwind flight and for flight duration in various male flies tested to food labelled by CS males.

The data correspond to those shown on Figure 6. The curves were analysed using Kaplan-Meier procedure with a subsequent Log-rank test and a post-hoc multiple pairs comparison (α=0.05 with Bonferroni correction); different letters beside the genotypes indicated in the legend reveals some significative differences between tester males. (a) upwind flight: $Khi^2_{(3df)} = 23.2$, $p < 10^{-4}$, (b) overall landing : $Khi^2_{(3df)} = 16.5$, $p < 10^{-4}$. For more information, please refer to Fig. S1.

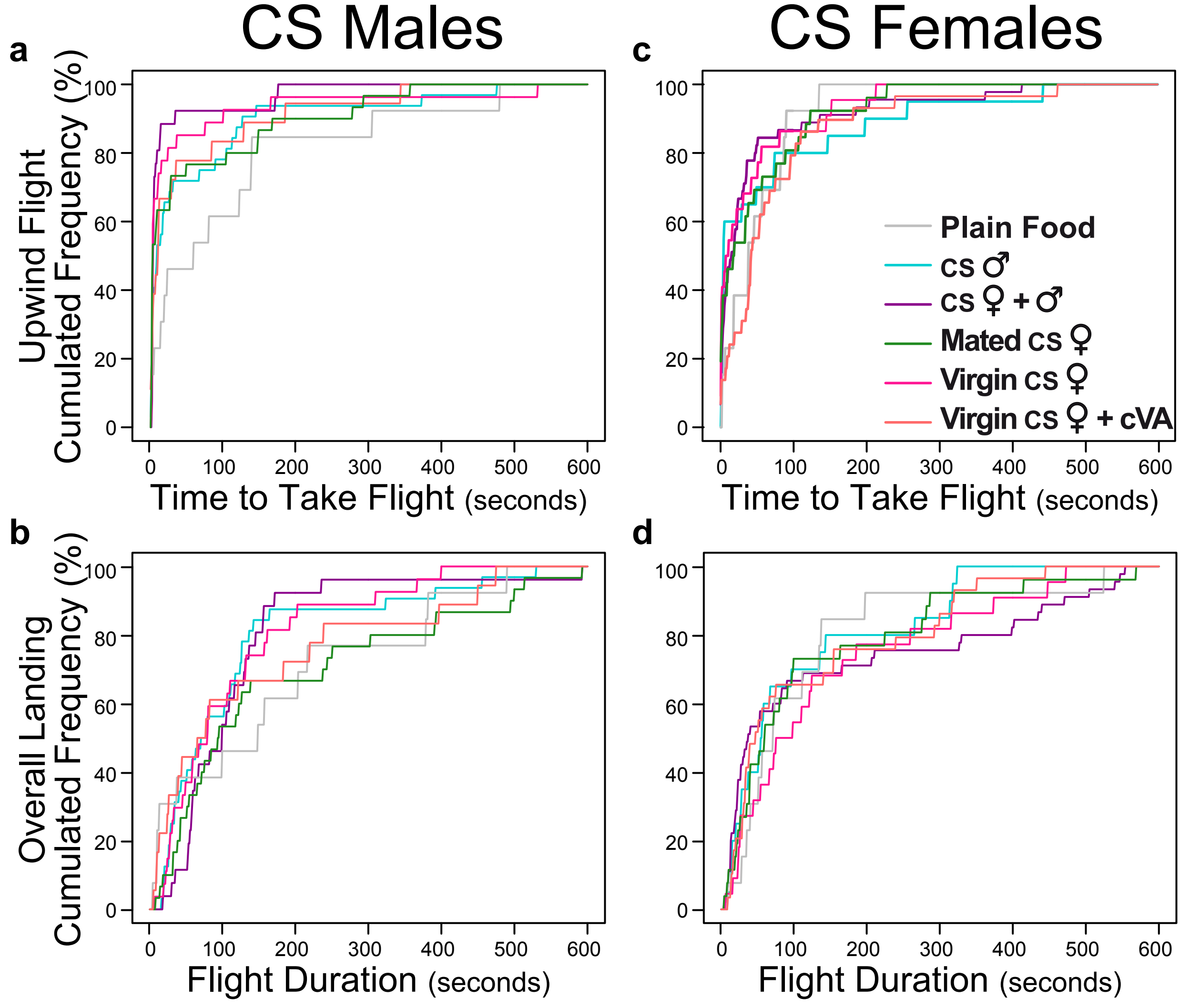

CS Males
CS Females
a
b
c
d
Upwind Flight
Cumulated Frequency (%)
Overall Landing
Cumulated Frequency (%)
Time to Take Flight (seconds)
Flight Duration (seconds)
0
20
40
60
80
100
0
100
200
300
400
500
600
Plain Food
CS ♂
CS ♀ + ♂
Mated CS ♀
Virgin CS ♀
Virgin CS ♀ + cVA

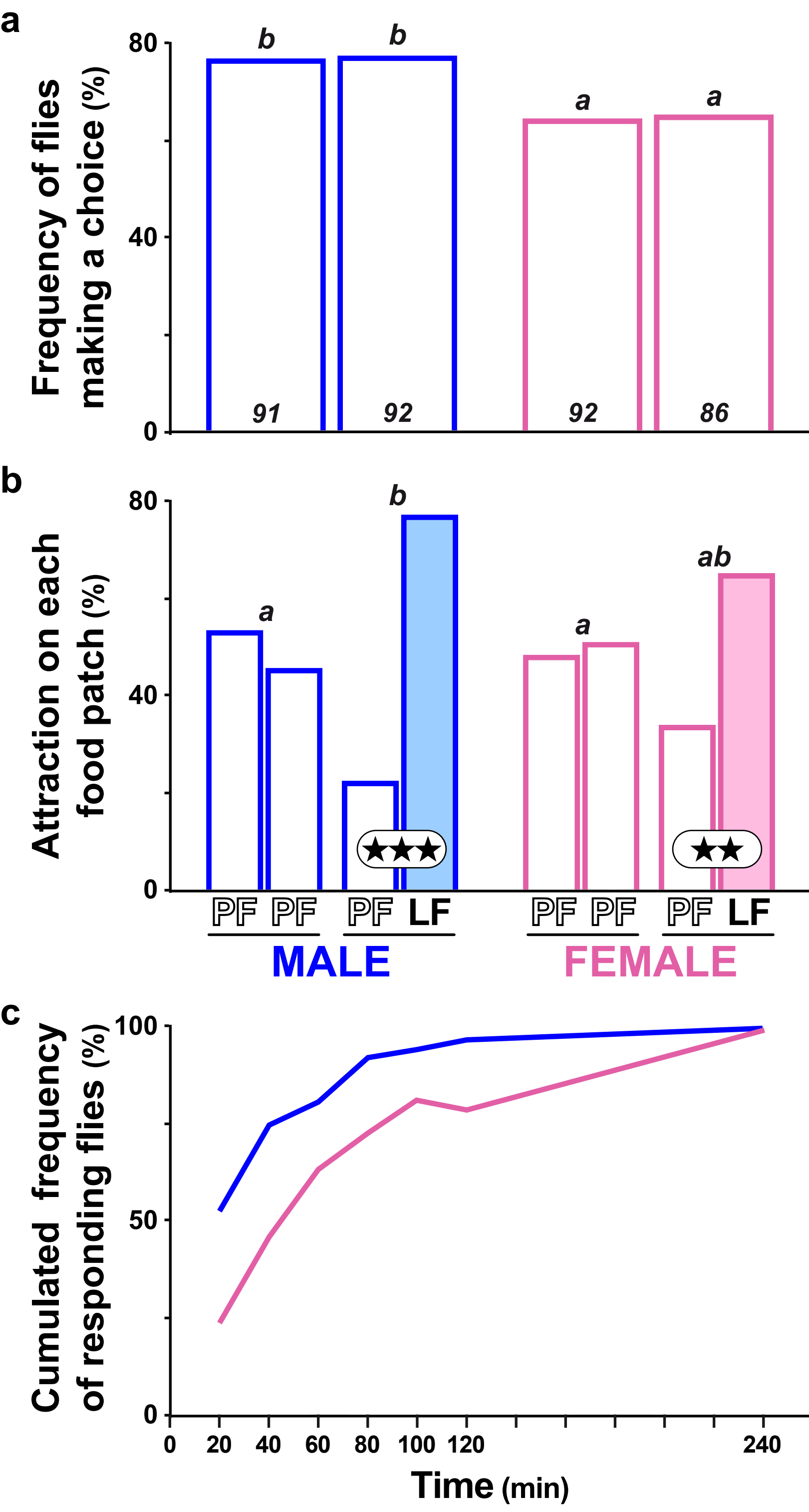

a
Frequency of flies making a choice (%)
80
40
0
b
b
a
a
91
92
92
86
b
Attraction on each food patch (%)
80
40
0
a
b
a
ab
★★★
★★
PF PF PF LF
PF PF PF LF
MALE
FEMALE
c
Cumulated frequency of responding flies (%)
100
50
0
0 20 40 60 80 100 120 240
Time (min)

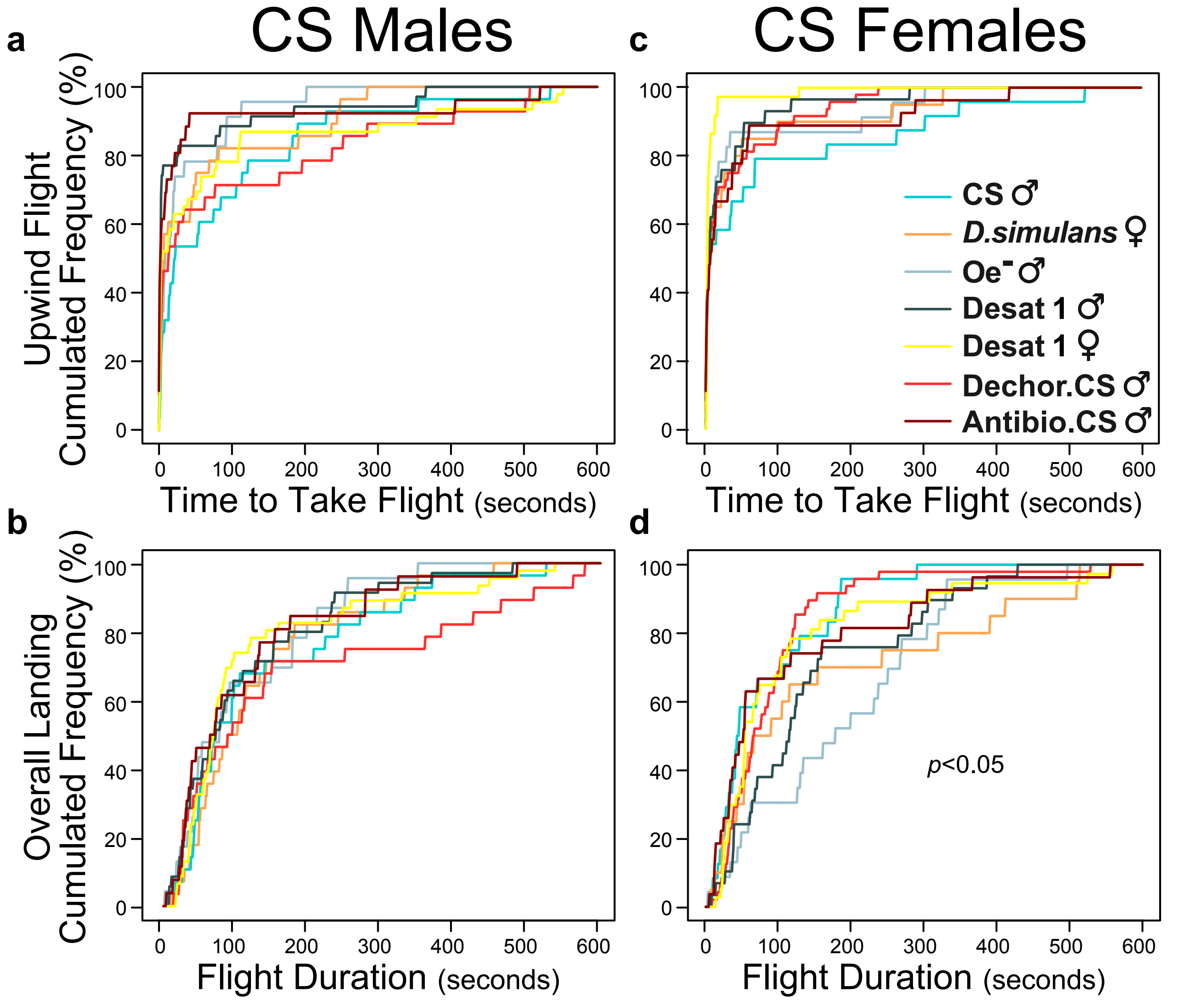

CS Males
CS Females
a
b
c
d
Upwind Flight
Cumulated Frequency (%)
Overall Landing
Cumulated Frequency (%)
Time to Take Flight (seconds)
Flight Duration (seconds)
0
20
40
60
80
100
0
100
200
300
400
500
600
CS ♂
D.simulans ♀
Oe⁻ ♂
Desat 1 ♂
Desat 1 ♀
Dechor.CS ♂
Antibio.CS ♂
p<0.05

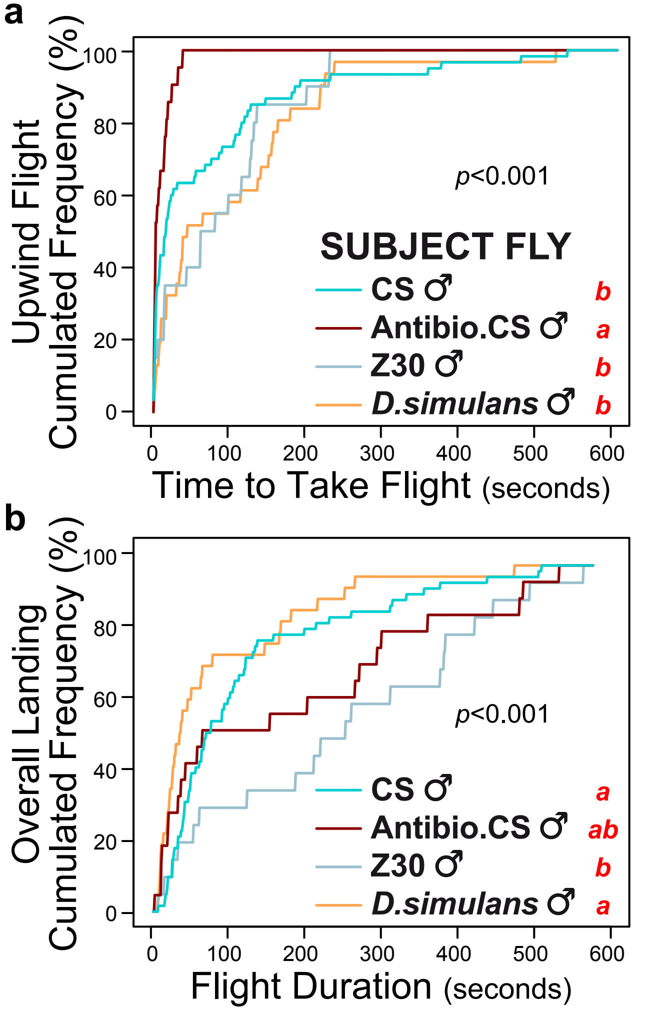
a
Upwind Flight Cumulated Frequency (%)
Time to Take Flight (seconds)
p<0.001
SUBJECT FLY
CS ♂ b
Antibio.CS ♂ a
Z30 ♂ b
D.simulans ♂ b
b
Overall Landing Cumulated Frequency (%)
Flight Duration (seconds)
p<0.001
CS ♂ a
Antibio.CS ♂ ab
Z30 ♂ b
D.simulans ♂ a
